# Atezolizumab-Induced Hypothyroidism in a Patient With Pre-existing Triiodothyronine (T3) Thyrotoxicosis Due to Graves’ Disease: A Case Report and Literature Review

**DOI:** 10.7759/cureus.19736

**Published:** 2021-11-19

**Authors:** Samson O Oyibo, Mohamed O Mahgoub

**Affiliations:** 1 Diabetes and Endocrinology, Peterborough City Hospital, Peterborough, GBR; 2 Oncology, Peterborough City Hospital, Peterborough, GBR

**Keywords:** t3-thyrotoxicosis, pre-existing, immune-checkpoint inhibitors, overt hypothyroidism, thyroiditis, graves´disease, triiodothyronine (t3), atezolizumab

## Abstract

The use of immune checkpoint inhibitors has improved the management and prognosis of many solid tumors. Because of their mechanism of action, and as checks on the immune systems are reduced, immune-related adverse events are common, including the exacerbation of the pre-existing autoimmune disease. The literature is scanty regarding reports of the use of immune checkpoint inhibitors in patients with pre-existing Graves’ disease. We report a case of a woman with pre-existing triiodothyronine (T3) thyrotoxicosis (hyperthyroidism) secondary to Graves’ disease, who developed thyroiditis followed by severe hypothyroidism after receiving an immune checkpoint inhibitor (atezolizumab) for the treatment of small-cell lung cancer. She had been on an anti-thyroid drug for Graves’ disease for two and a half years and was on the waiting list for a total thyroidectomy. However, the discovery of the severe hypothyroidism following atezolizumab-induced thyroiditis resulted in the need for long-term thyroid replacement therapy, and the planned surgery was no longer required. This case is one of the very few published reports of the use of atezolizumab in a patient with pre-existing Graves’ disease, resulting in the conversion from pre-existing T3-thyrotoxicosis to overt hypothyroidism. A multidisciplinary team approach is required when using immune checkpoint inhibitors in patients with pre-existing Graves’ disease or any other autoimmune disease.

## Introduction

Over the past decade, the use of immune checkpoint inhibitors has improved the management and prognosis of many solid tumors [1]. Immune checkpoints are receptors on the surface of immune cells that serve as breaks on the immune reaction. These include the programmed cell death 1 (PD-1) protein and its associated programmed cell death-ligand 1 (PD-L1) protein, and the cytotoxic T lymphocyte-associated protein 4 (CTLA-4). In the normal state, immune checkpoints help regulate the immune response, preventing inappropriate reactions and autoimmunity. However, tumor cells use this mechanism to evade the immune system. Cancer cells neutralize the immune system by halting immune cell activation through immune checkpoints. By using the immune system’s own regulatory mechanisms against it, cancer cells successfully escape the anti-cancer immune response. Immune checkpoint inhibitors are monoclonal antibodies that block the immune checkpoint proteins. Using monoclonal antibodies to block these immune checkpoint proteins, enables the immune system to mount an effective anti-cancer response [2,3]. The current immune checkpoint inhibitors include anti-programmed cell death 1 (anti-PD-1) agents (nivolumab and pembrolizumab), anti-programmed cell death-ligand 1 (anti-PD-L1) agents (atezolizumab, durvalumab and avelumab) and cytotoxic T lymphocyte-associated protein 4 inhibitors (anti-CTLA-4) like ipilimumab and tremelimumab [4].

Immune checkpoint inhibitors are associated with several immune-related adverse events (IRAE). Because of their mechanism of action, and as checks on the immune systems are reduced, the immune system is now free to attack other self-antigens while attacking the cancer cells [5]. Autoimmune endocrine gland dysfunction is among the commonest IRAE: the pituitary, thyroid, pancreas, adrenal and parathyroid glands can all be affected [6]. Thyroid gland dysfunction is the commonest type of endocrine IRAE that occurs during therapy with immune checkpoint inhibitors (especially the anti-PD-1 and anti-PD-L1 agents). The incidence rate is approximately 5%-8%: this could be as high as 14%-20% with combination therapy. Most patients developed destructive thyroiditis with an initial thyrotoxic phase followed by hypothyroidism, while very few patients developed Graves’ disease [7,8]. Thyroid abnormalities typically occur within one to three months after initiation of immune checkpoint inhibitor therapy. Treatment depends on whether there is thyrotoxicosis or hypothyroidism. Thyrotoxicosis may require beta-blockers for symptoms or rarely require anti-thyroid medication. Most cases of hypothyroidism require thyroid replacement therapy [8,9].

The occurrence of IRAE is more common in patients with pre-existing autoimmune disease than in patients without [9]. A flare-up of the pre-existing autoimmune disease is usually only mild to moderate and easily controlled by standard therapy [10]. However, the literature is scanty regarding reports of the use of immune checkpoint inhibitors in patients with pre-existing Graves’ disease.

We report a case of a woman with pre-existing T3-thyrotoxicosis secondary to Graves’ disease, who developed severe hypothyroidism after initiation of an immune checkpoint inhibitor (atezolizumab) for the treatment of small-cell lung cancer. This case emphasizes the importance of a multidisciplinary team approach when initiating immune checkpoint inhibitor therapy in patients with pre-existing autoimmune thyrotoxicosis or Graves’ disease.

## Case presentation

Medical history

A 58-year-old woman had her routine blood test, which indicated an exacerbation of her existing thyroid dysfunction. She had no new symptoms. Her medical history included triiodothyronine (T3) thyrotoxicosis (hyperthyroidism) secondary to Graves’ disease with stable thyroid eye disease, which had been going on for two and a half years. She was taking an anti-thyroid drug (carbimazole 5 mg daily) and was on the waiting list for a total thyroidectomy.

While on the waiting list for thyroidectomy, she had a routine ultrasound scan of her neck to assess for thyroid nodules. This revealed abnormally enlarged lymph nodes, which when histologically examined, unexpectedly exposed the presence of small cell lung cancer. Staging investigations revealed small cell lung cancer with mediastinal lymph nodes involvement and spread to the liver and adrenal gland, making it extensive-stage cancer. A week before the routine blood test revealing exacerbation of her thyroid dysfunction, she had received her first dose of intravenous immunotherapy (atezolizumab 1,200 mg) and intravenous chemotherapy (etoposide 148 mg and carboplatin 560 mg). She had a 40 pack-year history of smoking. She had no family history of thyroid dysfunction. There were no significant findings on clinical examination.

Investigations

**Figure 1:**
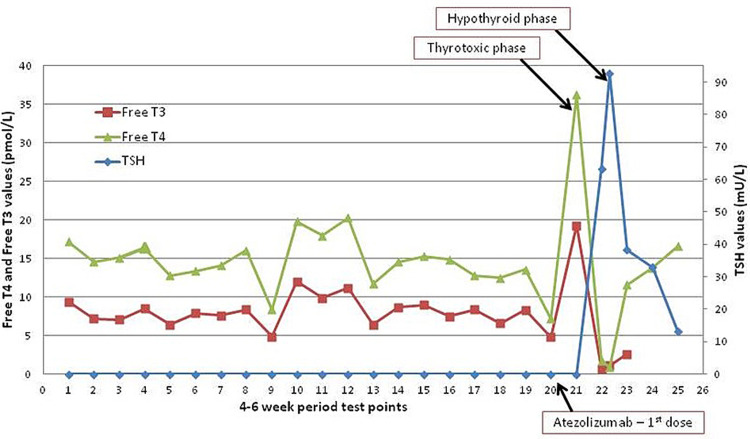
Graph showing thyroid function test results before and after the first dose of atezolizumab On the x-axis, each point represents a 4-6 week period. The left y-axis represents the free thyroxine (Free T4) and free triiodothyronine (Free T3) levels. The right y-axis represents the thyroid-stimulating hormone (TSH) levels. The point at which the first dose of atezolizumab was given, and the points at which the thyrotoxic phase and hypothyroid phase of atezolizumab-induced thyroiditis occurred are indicated.

Initial blood tests revealed mild leukocytosis, mild anemia and thrombocytopenia consistent with recent chemotherapy. The thyroid-stimulating hormone (TSH) level was as suppressed as usual, but the free triiodothyronine (Free T3) level was much higher than usual, and for the first time, the thyroxine (Free T4) level was elevated (Table 1). Thinking that this was just an exacerbation of her thyrotoxicosis, her carbimazole dose was increased to 20 mg daily. A repeat blood test four weeks later demonstrated severe hypothyroidism (TSH: 63.4 mU/L, Free T3: < 0.7 pmol/L, Free T4: 1.8 pmol/L), and markedly elevated anti-thyroid peroxidase antibody levels (>599 IU/mL), consistent with immune therapy-induced thyroiditis. The carbimazole was stopped, and a repeat blood test after two weeks demonstrated deterioration (TSH: 92.7 mU/L, Free T3: 2.6 pmol/L, Free T4: 0.9 pmol/L). Previous thyroid function test results indicated relatively stable T3-thyrotoxicosis for two and a half years prior to receiving the first dose of atezolizumab (Figure 1).

**Table 1:** Initial blood tests and results

Blood test	Result	Reference range
Sodium (mmol/L)	138	132-145
Potassium (mmol/L)	4.1	3.4-5.1
Chloride (mmol/L)	107	97-110
Creatinine (μmol/L)	53	45-84
Thyroid-stimulating hormone (mU/L)	0.01	0.3-4.2
Free thyroxine (pmol/L)	36.3	12.0-22.0
Free triiodothyronine (pmol/L)	19.3	3.1-6.8
Total protein (g/L)	68	60-80
Albumin (g/L)	40	35-50
Globulin (g/L)	28	20-35
Alanine transferase (U/L)	24	10-60
Alkaline phosphatase (U/L)	79	30-130
Haemoglobin (g/L)	95	115-165
White cell count (10^9^/L)	2.3	4.0-11.0
Platelet count (10^9^/L)	74	150-400

Treatment

The carbimazole was stopped and the thyroidectomy was no longer required. The patient started thyroid hormone replacement therapy, in the form of levothyroxine 100 mcg daily. The dose was gradually increased to 150 mcg daily after subsequent blood tests. She continued her immunotherapy and chemotherapy cycles.

Outcome and follow up

The patient continues on thyroid replacement therapy, which will probably be lifelong. The patient remains relatively well under both endocrinology and oncology outpatient follow-up.

## Discussion

Atezolizumab is a monoclonal antibody, which binds to the PD-L1, thereby reactivating the immune response to cancer cells. Atezolizumab has been used to treat urothelial carcinoma, non-small cell lung cancer, triple-negative breast cancer, small cell lung cancer, and hepatocellular carcinoma [11]. The National Institute for Health and Care Excellence (NICE) has recommended the use of immunotherapy in the form of atezolizumab in addition to chemotherapy in the form of carboplatin and etoposide as a treatment option for extensive-stage small-cell lung cancer making it the first-line treatment of choice [4].

The occurrence of thyroid IRAE, namely thyroiditis in patients treated with atezolizumab has been as high as 21% [12]. Ironically, studies have demonstrated that PD-L1 inhibitor-induced thyroiditis is associated with better overall survival in cancer patients. This interesting observation was related to atezolizumab both as monotherapy and in combination therapy [12]. There is scanty data regarding the use of atezolizumab in patients with pre-existing thyrotoxicosis or Graves’ disease.

Our patient had pre-existing T3-thyrotoxicosis. Thyrotoxicosis is a hyper-metabolic condition associated with elevated levels of Free T4 and/or Free T3 and low to undetectable TSH levels [13]. T3 thyrotoxicosis is characterized by elevated serum Free T3 levels, suppressed TSH levels, with normal Free T4 levels. This accounts for approximately 5% of cases of thyrotoxicosis. T3-thyrotoxicosis is caused by iodine deficiency or compensatory increased hormone production or faster peripheral T4 to T3 conversion or increased hormone production in patients with Graves’ disease, single toxic nodule or multinodular disease [14]. Graves’ disease is treated by decreasing thyroid hormone synthesis with the use of anti-thyroid drugs or by reducing the amount of thyroid tissue with radioactive iodine treatment or total thyroidectomy. Patients usually have medical treatment for the first 12-18 months with anti-thyroid drugs [13].

Our patient had been relatively stable on an anti-thyroid drug (carbimazole), before being placed on the waiting list for thyroidectomy. Her serum Free T3 levels were mainly mildly elevated, her serum TSH always suppressed, while her serum Free T4 levels had never been above the normal reference range. Therefore, the atezolizumab converted her condition from pre-existing Graves’ thyrotoxicosis to autoimmune hypothyroidism. The initial rise in her thyroid hormones likely represents the initial thyrotoxic phase of atezolizumab-induced thyroiditis. Our patient did not have a Doppler ultrasound scan to confirm reduced vascular flow associated with thyroiditis. Although thyroiditis followed by hypothyroidism is common with atezolizumab, there are limited published reports of this occurring in patients with pre-existing thyrotoxicosis.

There are retrospective pharmaceutical trial data concerning the use of immune checkpoint inhibitors in patients with other pre-existing autoimmune diseases. Data demonstrated that flare-ups of pre-existing autoimmune conditions are not common, they are manageable and rarely enough to discontinue treatment with immune checkpoint inhibitors [15,16]. A multidisciplinary team approach, involving the autoimmune disease specialist is important when initiating immune checkpoint inhibitor therapy in patients with pre-existing autoimmune diseases.

Reports concerning the use of immune checkpoint inhibitors in patients with Graves’ disease are scanty. One retrospective study found two patients with pre-existing Graves’ disease on an immune checkpoint inhibitor: one patient on nivolumab developed an exacerbation, while the other on pembrolizumab had no change in their thyroid status [16]. In another retrospective analysis of 19 patients with pre-existing thyrotoxicosis, 31.6% had no change, 52.6% had worsened and 15.8% had normalized (names of the immune checkpoint inhibitors were not mentioned in this report) [17]. Interestingly, there is a report of a patient with a previous history of Graves’ disease who developed severe thyroid eye disease while on ipilimumab [18]. In another case series, three patients had pre-existing Graves’ disease before starting an immune checkpoint inhibitor (two on nivolumab and one on pembrolizumab). All three of these patients developed hypothyroidism and came off their anti-thyroid drugs [19]. In another subgroup analysis of a large study using atezolizumab, only two patients had pre-existing Graves’ disease. The thyrotoxicosis normalized in one patient and remained ongoing in the other [20]. Therefore, our case is one of the very few published reports of the use of atezolizumab in a patient with pre-existing Graves’ disease, resulting in the conversion from pre-existing thyrotoxicosis to overt hypothyroidism.

## Conclusions

The use of immune checkpoint inhibitors in patients with pre-existing autoimmune disease can result in exacerbation or flare-up of that disease. Immune checkpoint inhibitors can exacerbate pre-existing Graves’ disease or convert it to autoimmune hypothyroidism, as described in this case report. A multidisciplinary team approach is required when using immune checkpoint inhibitors in patients with pre-existing autoimmune thyrotoxicosis or any other autoimmune diseases.
