# Correction: Atezolizumab-Induced Hypothyroidism in a Patient With Pre-existing Triiodothyronine (T3) Thyrotoxicosis Due to Graves’ Disease: A Case Report and Literature Review

**DOI:** 10.7759/cureus.c53

**Published:** 2021-11-22

**Authors:** Samson O Oyibo, Mohamed O Mahgoub

**Affiliations:** 1 Diabetes and Endocrinology, Peterborough City Hospital, Peterborough, GBR; 2 Oncology, Peterborough City Hospital, Peterborough, GBR

Shortly after publication, the submitting author realized that two statistical values in Table 1 had been entered incorrectly during the article submission process. As a result, we have corrected Table 1 to include the proper values as these were incorrectly transposed in the original version submitted to and published in Cureus:

Free thyroxine (pmol/L): 36.3

Free triiodothyronine (pmol/L): 19.3

